# Contrasting geographic structure in evolutionarily divergent Lake Tanganyika catfishes

**DOI:** 10.1002/ece3.3860

**Published:** 2018-02-06

**Authors:** Claire R. Peart, Kanchon K. Dasmahapatra, Julia J. Day

**Affiliations:** ^1^ Department of Genetics, Evolution and Environment University College London London UK; ^2^ Department of Life Sciences The Natural History Museum London UK; ^3^ Department of Biology University of York York UK; ^4^ Present address: Division of Evolutionary Biology Faculty of Biology, Ludwig‐Maximilians‐Universität München Planegg‐Martinsried Germany

**Keywords:** catfishes, diversification, East African Great Lakes, lake‐level fluctuations, Lake Tanganyika, Phylogeography, RADseq

## Abstract

Geographic isolation is suggested to be among the most important processes in the generation of cichlid fish diversity in East Africa's Great Lakes, both through isolation by distance and fluctuating connectivity caused by changing lake levels. However, even broad scale phylogeographic patterns are currently unknown in many non‐cichlid littoral taxa from these systems. To begin to address this, we generated restriction‐site‐associated DNA sequence (RADseq) data to investigate phylogeographic structure throughout Lake Tanganyika (LT) in two broadly sympatric rocky shore catfish species from independent evolutionary radiations with differing behaviors: the mouthbrooding claroteine, *Lophiobagrus cyclurus*, and the brood‐parasite mochokid, *Synodontis multipunctatus*. Our results indicated contrasting patterns between these species, with strong lake‐wide phylogeographic signal in *L. cyclurus* including a deep divergence between the northern and southern lake basins. Further structuring of these populations was observed across a heterogeneous habitat over much smaller distances. Strong population growth was observed in *L. cyclurus* sampled from shallow shorelines, suggesting population growth associated with the colonization of new habitats following lake‐level rises. Conversely, *S. multipunctatus*, which occupies a broader depth range, showed little phylogeographic structure and lower rates of population growth. Our findings suggest that isolation by distance and/or habitat barriers may play a role in the divergence of non‐cichlid fishes in LT, but this effect varies by species.

## 1 INTRODUCTION

The study of species diversification in biodiverse environments can reveal the importance of different factors influencing species diversity and coexistence. In the large East African rift lakes, multiple factors have been implicated in species divergence, for example, ecological adaptation, sexual selection, and allopatric divergence (Salzburger, Van Bocxlaer, & Cohen, 2014 and refs therein). Habitat breaks creating phylogeographically structured populations may provide the seed for future speciation and increased genetic diversity, but what constitutes a habitat break varies between species living in the same environment, as observed in cichlid fishes and their parasites (Baric, Salzburger, & Sturmbauer, 2003; Grégoir et al., 2015; Koblmüller et al., 2011; Sefc, Baric, Salzburger, & Sturmbauer, 2007; Van Oppen et al., 1997; Wagner & McCune, 2009). While mechanisms of diversification are intensively studied in these highly speciose radiations, the extent to which the observed geographic patterns apply to taxa from non‐cichlid radiations remains to be tested, with even broad scale geographic patterns poorly understood.

Lake Tanganyika (LT), the oldest African rift lake, contains exceptional species richness with high levels of endemism and multiple independent radiations (e.g., Day, Cotton, & Barraclough, 2008; Meyer, Matschiner, & Salzburger, 2015) even among non‐cichlid taxa, for example, Platytelphusid crabs (Marijnissen et al., 2006), multiple gastropod lineages (West & Michel, 2000; Wilson, Glaubrecht, & Meyer, 2004), and mastacembelid eels (Brown, Rüber, Bills, & Day, 2010). The elevated diversity of LT has been attributed in part to the lake's complex history, because it was formed from multiple basins varying in connectivity and size (Cohen, Soreghan, & Scholz, 1993). During the Late Pleistocene glaciations (≈106 Ka), lake levels dropped by ≈435 m, and while the size of LT was considerably reduced, it remained a large and mostly connected water body (McGlue et al., 2008). These lake‐level fluctuations have been shown to influence distributions and diversification in multiple cichlid species (e.g., Rüber, Verheyen, & Meyer, 1999; Sefc, Mattersdorfer, Hermann, & Koblmüller, 2017; Sturmbauer, Börger, van Steenberge, & Koblmüller, 2017; Verheyen, Rüber, Snoeks, & Meyer, 1996) primarily through altered habitat barriers and repeated periods of isolation followed by secondary contact (e.g., Egger, Koblmüller, Sturmbauer, & Sefc, 2007; Nevado, Mautner, Sturmbauer, & Verheyen, 2013). Lake‐level rises also correlate with population expansions allowing the colonization of new habitats (Koblmüller et al., 2011; Winkelmann, Rüber, & Genner, 2017). This complex history has influenced a range of geographic patterns in LT cichlids, as the environment interacts with species‐specific ecological characteristics, and population structure varies between even closely related species (e.g., Baric et al., 2003). Species‐specific characteristics play a large role, with some species showing little structure (e.g., Koblmüller, Odhiambo, Sinyinza, Sturmbauer, & Sefc, 2015), while in another species, a habitat break as small as 7 km acted as a barrier to dispersal (Sefc et al., 2007).

Lake Tanganyika catfishes (~34 species), as with cichlids from this lake, comprise multiple independent radiations (Day, Bills, & Friel, 2009; Day & Wilkinson, 2006; Peart, Bills, Wilkinson, & Day, 2014; Wright & Bailey, 2012) albeit on a smaller scale. They also share a similar habitat range, from deep to littoral waters, with littoral species vulnerable to displacement by lake‐level changes. Here, we focus on two species with lake‐wide distributions selected from independent evolutionary radiations, the claroteine (15 described LT species), *Lophiobagrus cyclurus*, and the mochokid (≈11 LT species), *Synodontis multipunctatus*. *L. cyclurus* is a mouthbrooder (Ochi, Rossiter, & Yanagisawa, 2002), a trait shared with many cichlid species, which may reduce dispersal ability through extended parental care, whereas *S. multipunctatus* is a known brood parasite of multiple cichlid species (Sato, 1986). Both species occur over rocky shores, although while *L. cyclurus* is thought to be restricted to the littoral zone (Bailey & Stewart, 1984), *S. multipunctatus* can also tolerate a greater depth range (sampled up to 170 m, Coulter, 1991). Fossil‐calibrated molecular dating indicates that both *L. cyclurus* and *S. multipunctatus* diverged from their sister species before the onset of climatic fluctuations in the Late Pleistocene (Day et al., 2013; Peart et al., 2014), suggesting that the present‐day geographic distributions of these species do not reflect the location of a recent origin.

The high sample number required for the techniques previously employed to investigate populations of LT taxa, for example, mitochondrial sequences, microsatellites (Koblmüller et al., 2015; Wagner & McCune, 2009), and AFLPs (Egger et al., 2007), has been a barrier to undertaking intraspecific studies on poorly sampled non‐cichlid taxa. Here, we use restriction‐site‐associated DNA sequencing (RADseq) to generate high numbers of loci per individual, allowing robust estimates of differentiation to be calculated using small numbers of individuals (e.g., Willing, Dreyer, & van Oosterhout, 2012). RAD loci were used to compare population structure in the two focal species to address the following questions: (1) Is there any evidence of lake‐wide phylogeographic structure? (2) Is there evidence of population growth at each site and is this more pronounced at the more recent shallower shorelines? (3) Is there localized population structure in *L. cyclurus* in areas with potential habitat barriers?

## 2 METHODS

**Figure 1:**
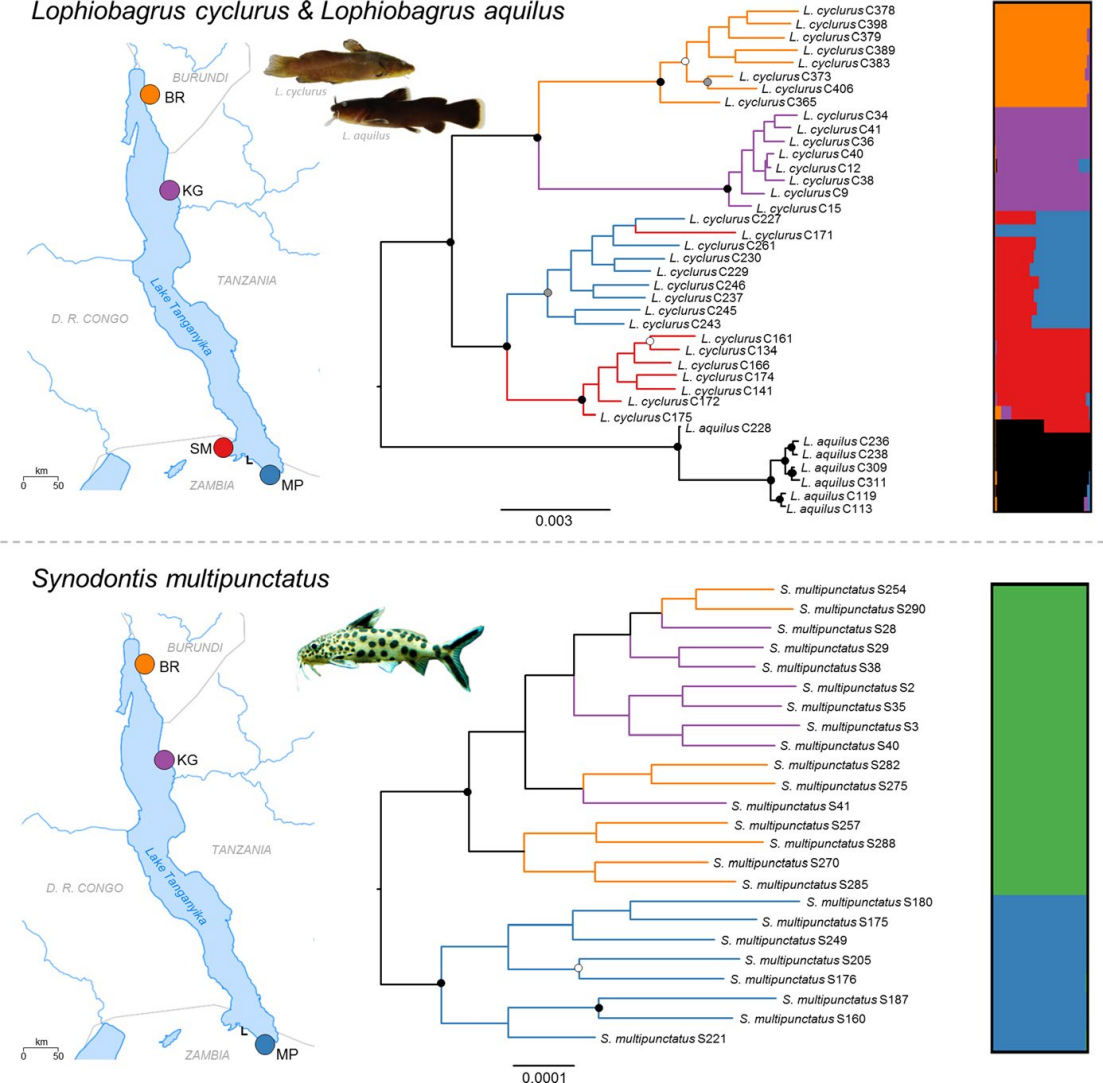
Maximum‐likelihood trees based on 2,628,515‐bp and 3,356,174‐bp alignments (bootstrap support: black circles 100%, gray circles >90%, white circles >80%) and structure plots, based on 23,739 SNPs and 16,443 SNPs for *Lophiobagrus* and *Synodontis multipunctatus,* respectively. Colors in the phylogeny depict collection locality. Green in the *S. multipunctatus* plot comprises sites from the northern basin. Sampling localities: BR ‐ Bujumbura Rural, KG—Kigoma SM—Sumbu, MP—Mpulungu. Major rivers are shown on the map, the inflow of the (smaller) Lufubu river is denoted with an “L”

A total of 57 individuals from the focal species (33 *L. cyclurus* and 24 *S. multipunctatus* specimens), sampled from four main locations around LT (Figure 1, Table S1), were included in this study. Samples were collected from one steeper shoreline (Kigoma) and three shallower shorelines (Bujumbura Rural, Mpulungu, Sumbu) (Figure 1 in Scholz et al., 2007). In addition, as the relationship between *L. cyclurus* and its hypothesized sister species, the paternal mouthbrooder *L*. *aquilus* (Ochi et al., 2002), has not previously been resolved (Peart et al., 2014), seven *L. aquilus* specimens from Zambia (Table S1) were also included. RADseq libraries were constructed following a protocol modified from Baird et al. (2008). Briefly, SbfI was used to digest the samples and each sample was individually barcoded. Samples were pooled into four libraries, each containing 16 samples, and size selection was then performed to select fragments 300–700 bp. The libraries were amplified using 17 PCR cycles and sequenced across two lanes of 100‐bp paired‐end Illumina Hi‐Seq2500 (two libraries per lane).

### 2.1 Bioinformatic processing

Bioinformatic processing was conducted as in Hoffman et al. (2014). In brief, the Stacks pipeline v1.08 (Catchen, Hohenlohe, Bassham, Amores, & Cresko, 2013) was used for demultiplexing and run using the forward reads following a superparent approach. The paired‐end reads for each locus were then assembled using Velvet (Zerbino & Birney, 2008) and used to generate a reference with the first read connected to the paired‐end by a string of Ns. All tags with more than one assembled contig were eliminated to avoid including instances where several tags had been collapsed into one tag in the clustering process, which ignores the second read. The *Lophiobagrus* reference consisted of 33,833 contigs with lengths ranging from 595 bp to 1,140 bp. The *S. multipunctatus* reference consisted of 26,472 contigs with lengths ranging from 592 bp to 1,133 bp. The Burrows–Wheeler algorithm was used to map each sample using BWA‐MEM (Li, 2013), duplicates were removed using picard‐tools 1.102 (http://picard.sourceforge.net), and SNPs were called using GATK UnifiedGenotyper (McKenna et al., 2010). The resultant vcf file was filtered to include only high‐quality calls with the following parameters; only biallelic SNPs, SNP quality score of 30, genotype quality score of 20, mapping quality score of 20, sites with coverage under five were excluded. In addition, in an attempt to avoid including repeated transposon regions in all datasets, sites with excessive coverage (the upper 5% of the coverage distribution) were excluded. Sites that were present in at least two individuals were included as it has been shown that stringent exclusion of missing data can bias the dataset (Huateng & Knowles, 2016). This resulted in very few called sites for the individual C137 from the *Lophiobagrus* dataset, and so, this individual was excluded from further analysis. The final *Lophiobagrus* dataset included 299,633 SNPs of which 266,434 SNPs were variable in *L. cyclurus*. The *S. multipunctatus* dataset consisted of 107,321 SNPs.

### 2.2 Population structure

Maximum‐likelihood (ML) trees were calculated with the GTR+GAMMA model using RAxML 7.7.8 (Stamatakis, 2006) with 1,000 nonparametric rapid bootstraps. These analyses were performed on alignments containing both variant and nonvariant sites: 2,628,515 sites for the *Lophiobagrus* dataset and 3,356,174 sites for the *S. multipunctatus* dataset. This analysis was also repeated using alignments with no missing data across individuals leading to 350,347 sites for the *Lophiobagrus* dataset and 1,479,271 sites for the *S. multipunctatus* dataset.

Population structure was investigated using Bayesian clustering in *structure* 2.3 (Pritchard, Stephens, & Donnelly, 2000) using a matrix of variable sites. *Structure* uses unlinked markers, so only one SNP per contig was retained, leading to datasets of 23,739 sites for *Lophiobagrus* and 16,443 for *S. multipunctatus*. Five replicates per *K* value (*K* 1–8) were run using 100,000 generations as burn‐in before sampling 100,000 generations using a model of admixture with correlated allele frequencies (Falush, Stephens, & Pritchard, 2003). Runs were collated using STRUCTURE HARVESTER (Earl & VonHoldt, 2011). Independent runs for the suggested *K* values were combined and averaged in CLUMPP (Jakobsson & Rosenberg, 2007) and the output visualized in distruct (Rosenberg, 2004). An additional analysis was repeated using datasets of variable sites present in all individuals using only one SNP per contig (1,938 *Lophiobagrus* and 5,116 *S. multipunctatus*). The package fineRADstructure (v. 0.2) (Malinsky, Trucchi, Lawson, & Falush, 2016) was also used to investigate structure in both the *Lophiobagrus* and *S. multipunctatus* datasets. These analyses were performed using loci with one to four SNPs in the forward read (using loci defined by Stacks), with no missing data, leading to datasets of 10,380 sites for *Lophiobagrus* and 13,028 for *S. multipunctatus*. Samples were assigned to populations using 100,000 iterations as burn‐in before sampling 100,000 iterations. The trees were built using 10,000 iterations and the output visualized using the fineradstructureplot.r and finestructurelibrary.r R scripts (http://cichlid.gurdon.cam.ac.uk/fineRADstructure.html).

Structure within *L. cyclurus* and *S. multipunctatus* datasets was further investigated using principal component analysis (PCA) in the R packages “adegenet” (Jombart, Devillard, Dufour, & Pontier, 2008) and ade4 (Dray & Dufour, 2007). PCAs were conducted on datasets of variable sites present in all individuals using only one SNP per contig (2,065 *L. cyclurus* and 5,116 *S. multipunctatus*). *F*_ST_ values were calculated using the index of Reich, Thangaraj, Patterson, Price, and Singh (2009) as this method performed well on small sample sizes in a recent simulation study (Willing et al., 2012). Analyses were completed using one SNP per contig with code modified from Rheindt, Fujita, Wilton, and Edwards (2013). 95% confidence intervals for these *F*_ST_ values were calculated by jackknifing.

Admixture between populations of *L. cyclurus* was investigated with *D*‐Statistics (Durand, Patterson, Reich, & Slatkin, 2011; Green et al., 2010) using each supported four‐taxon tree (*L. aquilus* as outgroup). The significance of the *D*‐statistic (from 0) is usually calculated by computing the standard error of the *D*‐statistic using a block jackknife approach over linkage groups. This approach was not possible in this case, as in the absence of a reference genome, the order of RAD contigs is unknown, so reliability was assessed by randomly subsampling the dataset 1,000 times including 99%, 95%, 90%, 80%, and 70% of the data and calculating the mean and standard error of the *D*‐statistic. These analyses were conducted using a modified version of a python script from Rheindt et al. (2013).

The statistical significance of the relationship between geographic distance and genetic distance was assessed using Mantel tests with 999 permutations in the R package ade4 (Dray & Dufour, 2007). Straight‐line distances between collection localities were calculated using the haversine method for calculating distances on the surface of a sphere. Distances along the lakeshore were calculated using ERSI ArcMap 10's Network Analyst package using the Detailed Water Bodies layer. Lakeshore distances were only calculated for *L. cyclurus* as this taxon was sampled from the littoral zone (<15 m). The analysis was performed using average genetic distance between collection localities as within location samples are pseudoreplicates. The uncorrected P‐distances were calculated using the R package ape (Paradis, Claude, & Strimmer, 2004).

### 2.3 Demography

Population trends in each population were estimated using a subset of the most variable loci by constructing Extended Bayesian Skyline Plots (EBSPs) in BEAST (Drummond, Suchard, Xie, & Rambaut, 2012). We followed the protocol of Trucchi et al. (2014) to avoid overparameterization when analyzing RADseq datasets. For each population, this analysis was performed on three independent subsamples of 50 loci with four SNPs in the forward read (using loci defined by Stacks). Due to a shortage of four SNP loci found in all individuals within a population, loci found in at least seven individuals were used for *S. multipunctatus* samples from Mpulungu, and loci found in at least six individuals were used for analyses on *L. cyclurus* samples from Kigoma and *S. multipunctatus* samples from Bujumbura Rural and Kigoma. Each analysis was run until convergence, with Tracer (Rambaut & Drummond, 2009) used to visualize convergence and effective population sizes. Sample C171 was excluded from this analysis due to its placement in the phylogeny and population structure analyses (Figure 1). In the absence of any rapidly evolving markers with a known mutation rate to calibrate this analysis, it was not possible to date demographic events.

## 3 RESULTS

### 3.1 Population structure

*Lophiobagrus cyclurus* is supported as monophyletic in the maximum‐likelihood tree (100%, Figure 1 and Figure S1), which was not observed in previous analyses (Peart et al., 2014), although the *structure* and FineRADstructure analysis (*K* = 5, Figures 1, S2 and S3) highlight an admixed sample, C228, which is derived almost equally from the *L. aquilus* and Sumbu clusters. All analyses show a clear separation between the northern and southern basins, as well as between Kigoma and Bujumbura Rural within the northern basin. In the southern basin, the two maximally supported clades correspond to collection locality, with a single exception (C171). This sample, from Sumbu, nests within the Mpulungu clade, and in the *structure,* analysis is comprised entirely of a cluster found in all Mpulungu samples (blue in Figure 1 and Figure S2). The cluster most strongly associated with Sumbu (red) is also seen in the remainder of the Mpulungu samples. The same patterns within *L. cyclurus* were observed in the *structure* analysis at *K* = 4 (Figure S4). This placement of C171 as close to but distinct from the Mpulungu samples is also supported by the FineRADstructure analysis (Figure S3). The samples from the southern basin are not supported as monophyletic in the maximum‐likelihood tree constructed with no missing data (Figure S1).

**Figure 2:**
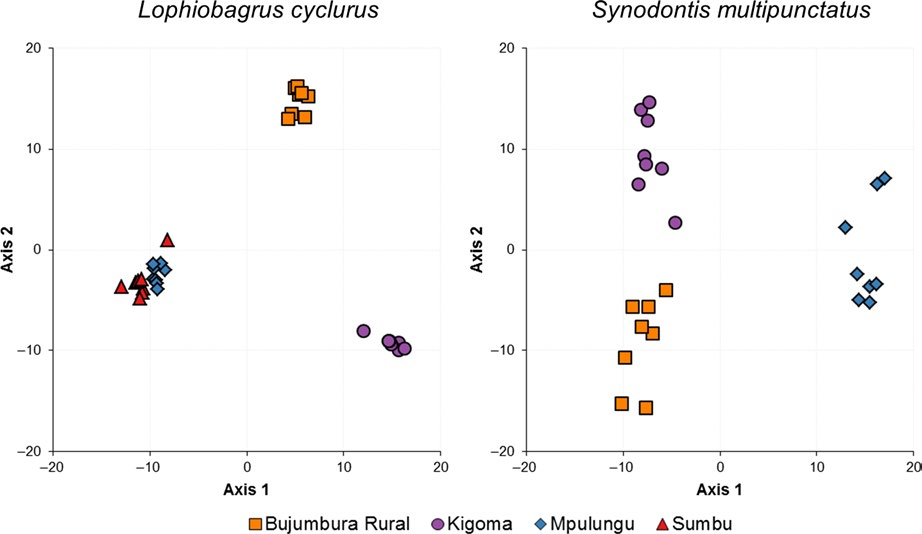
PCA plots using 2,065 and 5,116 SNPs for *Lophiobagrus cyclurus* and *Synodontis multipunctatus,* respectively. Colors depict collection locality

The population divisions in *L. cyclurus* are further supported by the PCA results (Figure 2) that show three distinct clusters relating to Kigoma, Bujumbura Rural, and the Zambian sites on the first two axes (19.3% and 13.7% of the variation, respectively). The third PCA axis also shows separation between the Zambian sites (Figure S5).

*F*_ST_ values between the Zambian populations and the population at Kigoma (0.191 [0.183–0.195, 95% confidence intervals], 0.198 [0.188–0.205]) were larger than those between the Zambian populations and the most northerly population (Bujumbura Rural) (0.162 [0.154–0.165], 0.170 [0.159–0.175]). Genetic distances (p‐distance) in *L*. *cyclurus* increased significantly with straight‐line distances (Mantel test *r* = 0.762, *p* = 0.004) and lake shore distances (Mantel test *r* = 0.759, *p* = 0.001) around LT. This significant relationship between genetic distance and straight‐line distance remained when only the Zambian samples were considered (Mantel test *r* = 0.482, *p* = 0.041) despite the low number of pairwise comparisons (not significant using lake shore distance, *r* = 0.479, *p* = 0.095). The *F*_ST_ value of 0.034 (0.024–0.040] indicates some differentiation at this smaller geographic scale.

*D*‐statistics indicated gene flow between all populations (Table 1) with greater gene flow between Kigoma and the Zambian sites than between Bujumbura and the Zambian sites, consistent with the larger distance between the latter sites. Subsampling of the data yielded similar values of the *D*‐statistic with standard errors that do not include zero. The *D*‐statistics increased in value when the analysis was repeated without the possible confounding sample C228 (Table S2).

**Table 1:** Nominal *D*‐statistics for *Lophiobagrus cyclurus* with mean and standard deviation from 1,000 random subsamples of the dataset at each percentage coverage. The taxa in bold show evidence of admixture based on the *D*‐statistics

Tree topology		Overall *D*‐statistic	*D*‐statistic ± standard deviation at subsampling level				
			99%	95%	90%	80%	70%
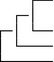	Burundi	0.0496	0.0496 (±1.2 × 10^−⁵^)	0.0497 (±4.1 × 10^−⁵^)	0.0496 (±4.6 × 10^−⁵^)	0.0497 (±1.1 × 10^−4^)	0.0495 (±7.6 × 10^−⁵^)
	**Kigoma**						
	**Mpulungu**						
	*L. aquilus*						
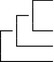	Burundi	0.0569	0.0569 (±1.1 × 10^−⁵^)	0.0569 (±2.9 × 10^−⁵^)	0.0568 (±7.1 × 10^−⁵^)	0.0570 (±8.3 × 10^−⁵^)	0.0570 (±8.2 × 10^−⁵^)
	**Kigoma**						
	**Sumbu**						
	*L. aquilus*						
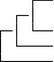	Mpulungu	0.0110	0.0110 (±1.1 × 10^−⁵^)	0.0110 (±2.3 × 10^−⁵^)	0.0110 (±3.7 × 10^−⁵^)	0.0110 (±8.6 × 10^−⁵^)	0.0109 (±6.3 × 10^−⁵^)
	**Sumbu**						
	**Burundi**						
	*L. aquilus*						
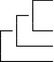	Mpulungu	0.0188	0.0188 (±1.5 × 10^−⁵^)	0.0188 (±7.8 × 10^−⁵^)	0.0188 (±5.2 × 10^−⁵^)	0.0188 (±4.5 × 10^−⁵^)	0.0190 (±6.4 × 10^−⁵^)
	**Sumbu**						
	**Kigoma**						
	*L. aquilus*						

In *S. multipunctatus* population structure was weaker. The maximum‐likelihood tree strongly supports the separation of populations from the northern and southern basins (100%) but finds no support for further structure within the northern basin. Support for the separation of the northern and southern basins is lower in the phylogeny built with no missing data (Figure S6). The *structure* analysis indicates a single population (*K* = 1); however, at *K* = 2, the northern and southern basins cluster separately (Figure 1 and Figure S7). The fineRADstructure analysis shows a clear separation of the northern and southern basins and also weaker structure within the northern basin (Figure S8). Similarly, the first two PCA axes represent 8.7% and 5.6% of the variation (Figure S9) and show three clusters corresponding to the sampled populations, although these are less tightly clustered than in *L. cyclurus* (Figure 2). The *F*_*ST*_ values were below zero, indicating a lack of population differentiation.

### 3.2 Demography

**Figure 3:**
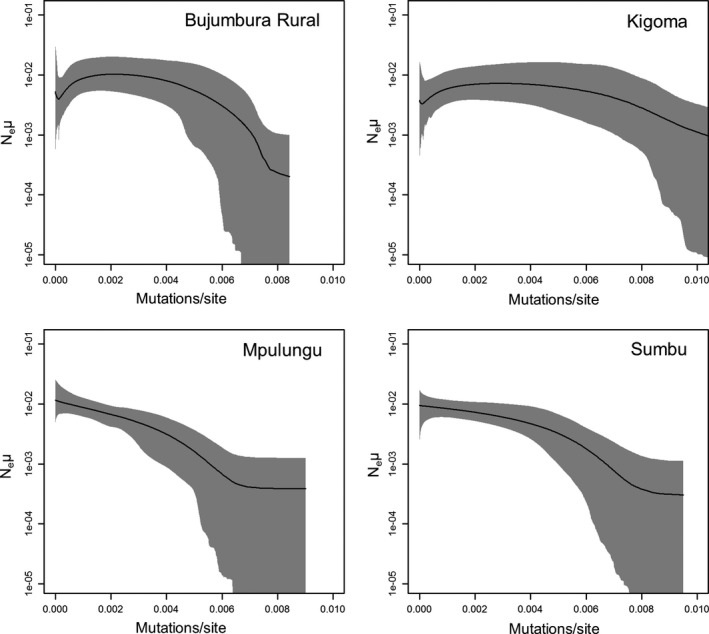
EBSPs for *Lophiobagrus cyclurus*. The y‐axis shows effective population size scaled by mutation rate (N_e_μ). Gray area represents 95% confidence interval

**Figure 4:**
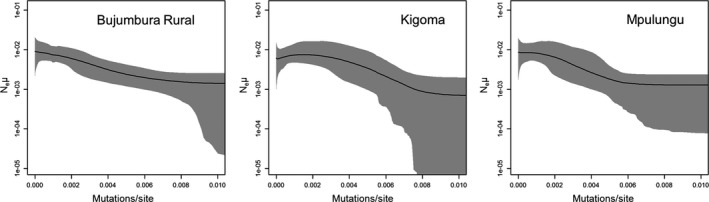
EBSPs for *Synodontis multipunctatus*. The *y*‐axis shows effective population size scaled by mutation rate (N_e_μ). Gray area represents 95% confidence interval

In general, mixing in the EBSP analyses was poor, requiring long run times (up to 2,150,000,000 generations). ESS values, with the exception of some operators, were above 200 in all analyses with the exception of one *S. multipunctatus* analysis from Kigoma and one from Mpulungu. It was not possible to continue these runs and so they were discarded. The null hypothesis of no population change could be rejected for each analysis with the exception of two *L. cyclurus* runs from Kigoma. The *L. cyclurus* results for Kigoma show either constant population or very weak growth, whereas the other three populations all show similar signatures of population growth (Figure 3 and Figure S10). All *S. multipunctatus* EBSP analyses show shallow population growth (Figure 4 and Figure S11). In Bujumbura Rural and Mpulungu, population growth is steeper in *L. cyclurus* than in *S. multipunctatus,* whereas both species show similar population trajectories in Kigoma.

## 4 DISCUSSION

Biodiversity hotspots contain a disproportionate amount of the world's diversity and as a result have received considerable attention regarding the patterns and processes underlying the generation and maintenance of species diversity. For the East African Great Lakes however, the vast majority of studies have focused on the hyperdiverse cichlid fishes, which may not exemplify the patterns of evolution seen in other fish groups, and it is not known whether the same factors influence other taxa that have diversified in these water bodies. To begin to remedy this knowledge gap, we focused on non‐cichlid taxa and show contrasting spatial patterns of phylogeographic structure in two broadly sympatric rocky shore catfish species from independent evolutionary radiations with different parental care strategies. We report strong lake‐wide population structure in *L. cyclurus*, including within basin differentiation. Conversely, population structure in *S. multipunctatus* is weak, and although there is some support for population differentiation between the northern and southern basins from the phylogenetic and structure analyses, this pattern is not reflected in the *F*_*ST*_ values suggesting that it is driven by a small number of SNPs. Such weak structure may be due to its dispersal ability at depth (Coulter, 1991). The only other LT species found at equivalent depths for which population structure has been investigated is the cichlid *Boulengerochromis microlepis* (Koblmüller et al., 2015) that showed similarly weak lake‐wide phylogeographic structure. The extent to which brood parasitism in *S. multipunctatus* (Sato, 1986) facilitates dispersal is unclear, because this species parasitizes both stenotypic and less geographically restricted species, for example, *Tropheus moorii*, (Sefc et al., 2007); *Simochromis diagramma* (Wagner & McCune, 2009).

At a smaller spatial scale, a high degree of genetic structure is observed between *L. cyclurus* populations in Sumbu and Mpulungu, which are only ~78 km apart. Previous cichlid studies have shown that river inflows and associated deltas can constitute barriers to gene flow (Wagner & McCune, 2009), suggesting a role for allopatry in increasing genetic diversity. This may also be a factor here because the Lufubu river inflow separates the Sumbu and Mpulungu sites. Microallopatry has been suggested as an important mechanism in generating diversity in cichlid fishes in African Great Lakes (e.g., Van Oppen et al., 1997) leading to the prediction that lineages showing increased genetic structure should also show higher species diversity. The evidence from *L*. *cyclurus* suggests that the endemic non‐cichlid LT radiations, while generally less diverse than the cichlid radiations, warrant finer‐scale investigations to determine the extent of population structure and diversity.

Populations in shallower lake regions are expected to be more affected by lake‐level fluctuations due to the availability of new habitat as lake levels rise and potential fusion of previously separated populations when lake levels fall. Our results from Kigoma (steeper shoreline) are consistent with the idea that populations are more stable at sites with steeper shorelines compared to shallow regions. However, there are more missing data in the reconstructions from Kigoma and we cannot discount the possibility that the weak or no population growth seen in *L. cyclurus* might be related to a lack of resolution to accurately reconstruct the demographic history. Without an absolute calibration of the EBSPs, it is not possible to date the reconstructions; however, the signatures of population growth at both Zambian sites in *L. cyclurus* are consistent with results from several cichlid species where populations grew following the most recent low stand as new habitat became available (Koblmüller et al., 2011; Winkelmann et al., 2017). *S. multipunctatus* showed weaker population growth than *L. cyclurus*, suggesting that because of its greater depth range, its demographic history has been less influenced by past lake‐level fluctuations.

The placement in both the phylogenetic and structure analyses of *L. aquilus*‐C228 suggested possible interbreeding between this species and *L. cyclurus*. Notably, this specimen was collected in Mpulungu, despite its partial assignment to a genetic cluster most common in the Sumbu *L. cyclurus* population and was identified as *L. aquilus* based on the key of Bailey and Stewart (1984). Within *L. cyclurus,* there was admixture between all populations with the overall patterns reflecting geographic structure. Population subdivision in ancestral species can also influence gene trees and the relative proportion of ABBA/BABA SNPs (Eriksson & Manica, 2012). It is possible that this affected our results as population structure with *L*. *aquilus* has not been investigated and this species was sampled only from Zambia.

### 4.1 Conclusions and future directions

Investigating diversity patterns both within and between species in biodiversity hotpots allow conclusions to be drawn as to which mechanisms are responsible for such elevated diversity. Here, we report contrasting geographic structure at a broad spatial scale in evolutionary divergent LT catfishes using genomic data. Our study suggests that lake‐level fluctuations have a role in structuring their diversity, although the genomic consequences of these differing histories remain to be studied. We recommend the study of additional species from non‐cichlid radiations with different ecologies to provide a more comprehensive understanding of evolutionary patterns and processes in these systems. Within LT catfishes restricted to the littoral zone, and in *L. cyclurus* in particular, fine‐scale population structure warrants further investigation with additional sampling localities required. This study is limited by low sample sizes, and further sampling would allow the timing and extent of gene flow between populations to be investigated plus the identification of habitat barriers for this species. Additional sampling localities in the southern basin of LT may allow the placement of sample C171 to be further understood in a broader geographic context. Further *L. aquilus* samples from these localities would also allow estimates of the extent of gene flow between these species to be investigated.

## ETHICS

Conducted under COSTECH permit: no. 2010‐03‐NA‐2009‐207.

## DATA ACCESSIBILITY

Sequence data are accessible on NCBI under SRP126168. Datasets used in the analyses are available on Dryad http://doi.org/10.5061/dryad.671bs

## CONFLICT OF INTEREST

None declared.

## AUTHOR CONTRIBUTION

All authors contributed to study design and wrote the manuscript. CRP and JJD conducted fieldwork, and CRP and KKD generated and analyzed the RAD data.

## References

[ece33860-bib-0001] Bailey, R. M. , & Stewart, D. J. (1984). Bagrid catfishes from Lake Tanganyika, with a key and descriptions of New Taxa (p. 168). Ann Arbor, MI: Misc. Publ. Museum Zool. Univ.

[ece33860-bib-0002] Baird, N. A. , Etter, P. D. , Atwood, T. S. , Currey, M. C. , Shiver, A. L. , Lewis, Z. A. , … Johnson, E. A. (2008). Rapid SNP discovery and genetic mapping using sequenced RAD markers. PLoS One, 3, e3376 https://doi.org/10.1371/journal.pone.0003376 18852878 10.1371/journal.pone.0003376 PMC2557064

[ece33860-bib-0003] Baric, S. , Salzburger, W. , & Sturmbauer, C. (2003). Phylogeography and evolution of the Tanganyikan cichlid genus *Tropheus* based upon mitochondrial DNA sequences. Journal of Molecular Evolution, 56, 54–68. https://doi.org/10.1007/s00239-002-2380-7 12569423 10.1007/s00239-002-2380-7

[ece33860-bib-0004] Brown, K. J. , Rüber, L. , Bills, R. , & Day, J. J. (2010). Mastacembelid eels support Lake Tanganyika as an evolutionary hotspot of diversification. BMC Evolutionary Biology, 10, 188 https://doi.org/10.1186/1471-2148-10-188 20565906 10.1186/1471-2148-10-188 PMC2903574

[ece33860-bib-0005] Catchen, J. , Hohenlohe, P. A. , Bassham, S. , Amores, A. , & Cresko, W. A. (2013). Stacks: An analysis tool set for population genomics. Molecular Ecology, 22, 3124–3140. https://doi.org/10.1111/mec.12354 23701397 10.1111/mec.12354 PMC3936987

[ece33860-bib-0006] Cohen, A. S. , Soreghan, M. J. , & Scholz, C. A. (1993). Estimating the age of formation of lakes: An example from Lake Tanganyika, East African Rift system. Geology, 21, 511 https://doi.org/10.1130/0091-7613(1993)021<0511:etaofo>2.3.co;2

[ece33860-bib-0007] Coulter, G. W. (1991). Lake Tanganyika and its life. Oxford, UK: Oxford University Press.

[ece33860-bib-0008] Day, J. J. , Bills, R. , & Friel, J. P. (2009). Lacustrine radiations in African *Synodontis* catfish. Journal of Evolutionary Biology, 22, 805–817. https://doi.org/10.1111/j.1420-9101.2009.01691.x 19226415 10.1111/j.1420-9101.2009.01691.x

[ece33860-bib-0009] Day, J. J. , Cotton, J. A. , & Barraclough, T. G. (2008). Tempo and mode of diversification of lake Tanganyika cichlid fishes. PLoS One, 3, e1730 https://doi.org/10.1371/journal.pone.0001730 18320049 10.1371/journal.pone.0001730 PMC2248707

[ece33860-bib-0010] Day, J. J. , Peart, C. R. , Brown, K. J. , Friel, J. P. , Bills, R. , & Moritz, T. (2013). Continental Diversification of an African Catfish Radiation (Mochokidae: Synodontis). Systematic Biology, 62, 351–365. https://doi.org/10.1093/sysbio/syt001 23302956 10.1093/sysbio/syt001

[ece33860-bib-0011] Day, J. J. , & Wilkinson, M. (2006). On the origin of the Synodontis catfish species flock from Lake Tanganyika. Biology Letter, 2, 548–552. https://doi.org/10.1098/rsbl.2006.0532 10.1098/rsbl.2006.0532 PMC1833983 17148285

[ece33860-bib-0012] Dray, S. , & Dufour, A. B. (2007). The ade4 Package: Implementing the Duality Diagram for Ecologists. Journal of Statistical Software, 22, 1–20.

[ece33860-bib-0013] Drummond, A. J. , Suchard, M. A. , Xie, D. , & Rambaut, A. (2012). Bayesian phylogenetics with BEAUti and the BEAST 1.7. Molecular Biology and Evolution, 29, 1969–1973. https://doi.org/10.1093/molbev/mss075 22367748 10.1093/molbev/mss075 PMC3408070

[ece33860-bib-0014] Durand, E. Y. , Patterson, N. , Reich, D. , & Slatkin, M. (2011). Testing for ancient admixture between closely related populations. Molecular Biology and Evolution, 28, 2239–2252. https://doi.org/10.1093/molbev/msr048 21325092 10.1093/molbev/msr048 PMC3144383

[ece33860-bib-0015] Earl, D. A. , & VonHoldt, B. M. (2011). STRUCTURE HARVESTER: A website and program for visualizing STRUCTURE output and implementing the Evanno method. Conservation Genetics Resources, 4, 359–361. https://doi.org/10.1007/s12686-011-9548-7

[ece33860-bib-0016] Egger, B. , Koblmüller, S. , Sturmbauer, C. , & Sefc, K. M. (2007). Nuclear and mitochondrial data reveal different evolutionary processes in the Lake Tanganyika cichlid genus *Tropheus* . BMC Evolutionary Biology, 7, 137 https://doi.org/10.1186/1471-2148-7-137 17697335 10.1186/1471-2148-7-137 PMC2000897

[ece33860-bib-0017] Eriksson, A. , & Manica, A. (2012). Effect of ancient population structure on the degree of polymorphism shared between modern human populations and ancient hominins. Proceedings of the National Academy of Sciences of the United States of America, 109, 13956–13960. https://doi.org/10.1073/pnas.1200567109 22893688 10.1073/pnas.1200567109 PMC3435202

[ece33860-bib-0018] Falush, D. , Stephens, M. , & Pritchard, J. K. (2003). Inference of population structure using multilocus genotype data: Linked loci and correlated allele frequencies. Genetics, 164, 1567–1587. 12930761 10.1093/genetics/164.4.1567 PMC1462648

[ece33860-bib-0019] Green, R. E. , Krause, J. , Briggs, A. W. , Maricic, T. , Stenzel, U. , Kircher, M. , … Pääbo, S. (2010). A draft sequence of the Neandertal genome. Science, 328, 710–722. https://doi.org/10.1126/science.1188021 20448178 10.1126/science.1188021 PMC5100745

[ece33860-bib-0020] Grégoir, A. F. , Hablützel, P. I. , Vanhove, M. P. M. , Pariselle, A. , Bamps, J. , Volckaert, F. A. M. , & Raeymaekers, J. A. M. (2015). A link between host dispersal and parasite diversity in two sympatric cichlids of Lake Tanganyika. Freshwater Biology, 60, 323–335. https://doi.org/10.1111/fwb.12492

[ece33860-bib-0021] Hoffman, J. I. , Simpson, F. , David, P. , Rijks, J. M. , Kuiken, T. , Thorne, M. A. S. , … Dasmahapatra, K. K. (2014). High‐throughput sequencing reveals inbreeding depression in a natural population. Proceedings of the National Academy of Sciences of the United States of America, 111, 3775–3780. https://doi.org/10.1073/pnas.1318945111 24586051 10.1073/pnas.1318945111 PMC3956162

[ece33860-bib-0022] Huateng, H. , & Knowles, L. (2016). Unforeseen consequences of excluding missing data from next‐generation sequences: Simulation study of rad sequences. Systematic Biology, 65, 357–365. https://doi.org/10.1093/sysbio/syu046 24996413 10.1093/sysbio/syu046

[ece33860-bib-0023] Jakobsson, M. , & Rosenberg, N. A. (2007). CLUMPP: A cluster matching and permutation program for dealing with label switching and multimodality in analysis of population structure. Bioinformatics, 23, 1801–1806. https://doi.org/10.1093/bioinformatics/btm233 17485429 10.1093/bioinformatics/btm233

[ece33860-bib-0024] Jombart, T. , Devillard, S. , Dufour, A.‐B. , & Pontier, D. (2008). Revealing cryptic spatial patterns in genetic variability by a new multivariate method. Heredity (Edinb), 101, 92–103. https://doi.org/10.1038/hdy.2008.34 18446182 10.1038/hdy.2008.34

[ece33860-bib-0025] Koblmüller, S. , Odhiambo, E. A. , Sinyinza, D. , Sturmbauer, C. , & Sefc, K. M. (2015). Big fish, little divergence: Phylogeography of Lake Tanganyika's giant cichlid, *Boulengerochromis microlepis* . Hydrobiologia, 748, 29–38. https://doi.org/10.1007/s10750-014-1863-z 25983338 10.1007/s10750-014-1863-z PMC4430823

[ece33860-bib-0026] Koblmüller, S. , Salzburger, W. , Obermüller, B. , Eigner, E. , Sturmbauer, C. , & Sefc, K. M. (2011). Separated by sand, fused by dropping water: Habitat barriers and fluctuating water levels steer the evolution of rock‐dwelling cichlid populations in Lake Tanganyika. Molecular Ecology, 20, 2272–2290. https://doi.org/10.1111/j.1365-294X.2011.05088.x 21518059 10.1111/j.1365-294X.2011.05088.x

[ece33860-bib-0027] Li, H . (2013) Aligning sequence reads, clone sequences and assembly contigs with BWA‐MEM. arXiv:1303.3997v1 [q‐bio.GN].

[ece33860-bib-0028] Malinsky, M. , Trucchi, E. , Lawson, D. , & Falush, D . (2016). RADpainter and fineRADstructure: Population inference from RADseq data. bioRxiv, https://doi.org/10.1101/057711 10.1093/molbev/msy023 PMC5913677 29474601

[ece33860-bib-0029] Marijnissen, S. A. E. , Michel, E. , Daniels, S. R. , Erpenbeck, D. , Menken, S. B. J. , & Schram, F. R. (2006). Molecular evidence for recent divergence of Lake Tanganyika endemic crabs (Decapoda: Platythelphusidae). Molecular Phylogenetics and Evolution, 40, 628–634. https://doi.org/10.1016/j.ympev.2006.03.025 16647274 10.1016/j.ympev.2006.03.025

[ece33860-bib-0030] McGlue, M. M. , Lezzar, K. E. , Cohen, A. S. , Russell, J. M. , Tiercelin, J.‐J. , Felton, A. A. , … Nkotagu, H. H. (2008). Seismic records of late Pleistocene aridity in Lake Tanganyika, tropical East Africa. J Paleolimnol, 40, 635–653. https://doi.org/10.1007/s10933-007-9187-x

[ece33860-bib-0031] McKenna, A. , Hanna, M. , Banks, E. , Sivachenko, A. , Cibulskis, K. , Kernytsky, A. , … DePristo, M. A. (2010). The Genome Analysis Toolkit: A MapReduce framework for analyzing next‐generation DNA sequencing data. Genome Research, 20, 1297–1303. https://doi.org/10.1101/gr.107524.110.20 20644199 10.1101/gr.107524.110 PMC2928508

[ece33860-bib-0032] Meyer, B. S. , Matschiner, M. , & Salzburger, W. (2015). A tribal level phylogeny of Lake Tanganyika cichlid fishes based on a genomic multi‐marker approach. Molecular Phylogenetics and Evolution, 83, 56–71. https://doi.org/10.1016/j.ympev.2014.10.009 25433288 10.1016/j.ympev.2014.10.009 PMC4334724

[ece33860-bib-0033] Nevado, B. , Mautner, S. , Sturmbauer, C. , & Verheyen, E. (2013). Water‐level fluctuations and metapopulation dynamics as drivers of genetic diversity in populations of three Tanganyikan cichlid fish species. Molecular Ecology, 22, 3933–3948. https://doi.org/10.1111/mec.12374 23837841 10.1111/mec.12374 PMC3763204

[ece33860-bib-0034] Ochi, H. , Rossiter, A. , & Yanagisawa, Y. (2002). Paternal mouthbrooding bagrid catfishes in Lake Tanganyika. Ichthyological Research, 49, 270–273. https://doi.org/10.1007/s102280200039

[ece33860-bib-0035] Paradis, E. , Claude, J. , & Strimmer, K. (2004). APE: Analyses of Phylogenetics and Evolution in R language. Bioinformatics, 20, 289–290. https://doi.org/10.1093/bioinformatics/btg412 14734327 10.1093/bioinformatics/btg412

[ece33860-bib-0036] Peart, C. R. , Bills, R. , Wilkinson, M. , & Day, J. J. (2014). Nocturnal claroteine catfishes reveal dual colonisation but a single radiation in Lake Tanganyika. Molecular Phylogenetics and Evolution, 73, 119–128. https://doi.org/10.1016/j.ympev.2014.01.013 24503480 10.1016/j.ympev.2014.01.013

[ece33860-bib-0037] Pritchard, J. K. , Stephens, M. , & Donnelly, P. (2000). Inference of population structure using multilocus genotype data. Genetics, 155, 945–959. 10835412 10.1093/genetics/155.2.945 PMC1461096

[ece33860-bib-0038] Rambaut, A. , & Drummond, A. J . (2009). Tracer v1.5. Retrieved from http://tree.bio.ed.ac.uk/software/tracer/.

[ece33860-bib-0039] Reich, D. , Thangaraj, K. , Patterson, N. , Price, A. L. , & Singh, L. (2009). Reconstructing Indian population history. Nature, 461, 489–494. https://doi.org/10.1038/nature08365 19779445 10.1038/nature08365 PMC2842210

[ece33860-bib-0040] Rheindt, F. E. , Fujita, M. K. , Wilton, P. R. , & Edwards, S. V. (2013). Introgression and Phenotypic Assimilation in *Zimmerius* Flycatchers (Tyrannidae): Population Genetic and Phylogenetic Inferences from Genome‐Wide SNPs. Systematic Biology, 1–19. https://doi.org/10.1093/sysbio/syt070 10.1093/sysbio/syt070 24304652

[ece33860-bib-0041] Rosenberg, N. A. (2004). Distruct: A program for the graphical display of population structure. Molecular Ecology Notes, 4, 137–138. https://doi.org/10.1046/j.1471-8286.2003.00566.x

[ece33860-bib-0042] Rüber, L. , Verheyen, E. , & Meyer, A. (1999). Replicated evolution of trophic specializations in an endemic cichlid fish lineage from Lake Tanganyika. Proceedings of the National Academy of Sciences of the United States of America, 96, 10230–10235. https://doi.org/10.1073/pnas.96.18.10230 10468591 10.1073/pnas.96.18.10230 PMC17871

[ece33860-bib-0043] Salzburger, W. , Van Bocxlaer, B. , & Cohen, A. S. (2014). Ecology and Evolution of the African Great Lakes and Their Faunas. Annual Review of Ecology, Evolution, and Systematics, 45, 519–545. https://doi.org/10.1146/annurev-ecolsys-120213-091804

[ece33860-bib-0044] Sato, T. (1986). A brood parasitic catfish of mouthbrooding cichlid fishes in Lake Tanganyika. Nature, 323, 58–59. https://doi.org/10.1038/323058a0 3748180 10.1038/323058a0

[ece33860-bib-0045] Scholz, C. A. , Johnson, T. C. , Cohen, A. S. , King, J. W. , Peck, J. A. , Overpeck, J. T. , … Pierson, J. (2007). East African megadroughts between 135 and 75 thousand years ago and bearing on early‐modern human origins. Proceedings of the National Academy of Sciences, 104, 16416–16421. https://doi.org/10.1073/pnas.0703874104 10.1073/pnas.0703874104 PMC1964544 17785420

[ece33860-bib-0046] Sefc, K. M. , Baric, S. , Salzburger, W. , & Sturmbauer, C. (2007). Species‐specific population structure in rock‐specialized sympatric cichlid species in Lake Tanganyika, East Africa. Journal of Molecular Evolution, 64, 33–49. https://doi.org/10.1007/s00239-006-0011-4 17160645 10.1007/s00239-006-0011-4

[ece33860-bib-0047] Sefc, K. M. , Mattersdorfer, K. , Hermann, C. M. , & Koblmüller, S. (2017). Past lake shore dynamics explain present pattern of unidirectional introgression across a habitat barrier. Hydrobiologia, 791, 69–82. https://doi.org/10.1007/s10750-016-2791-x 10.1007/s10750-016-2791-x PMC6557712 31186578

[ece33860-bib-0048] Stamatakis, A. (2006). RAxML‐VI‐HPC: Maximum likelihood‐based phylogenetic analyses with thousands of taxa and mixed models. Bioinformatics, 22, 2688–2690. https://doi.org/10.1093/bioinformatics/btl446 16928733 10.1093/bioinformatics/btl446

[ece33860-bib-0049] Sturmbauer, C. , Börger, C. , van Steenberge, M. , & Koblmüller, S. (2017). A separate lowstand lake at the northern edge of Lake Tanganyika? Evidence from phylogeographic patterns in the cichlid genus *Tropheus* . Hydrobiologia, 791, 51–68. https://doi.org/10.1007/s10750-016-2939-8

[ece33860-bib-0050] Trucchi, E. , Gratton, P. , Whittington, J. D. , Cristofari, R. , Le Maho, Y. , Stenseth, N. C. , & Le Bohec, C. (2014). King penguin demography since the last glaciation inferred from genome‐wide data. Proceedings Biological Science., 281, 20140528 https://doi.org/10.1098/rspb.2014.0528 10.1098/rspb.2014.0528 PMC4071544 24920481

[ece33860-bib-0051] Van Oppen, M. J. H. , Turner, G. F. , Rico, C. , Deutsch, J. C. , Ibrahim, K. M. , Robinson, R. L. , & Hewitt, G. M. (1997). Unusually fine‐scale genetic structuring found in rapidly speciating Malawi cichlid fishes. Proceedings of the Royal Society B: Biological Science, 264, 1803–1812. https://doi.org/10.1098/rspb.1997.0248

[ece33860-bib-0052] Verheyen, E. , Rüber, L. , Snoeks, J. , & Meyer, A. (1996). Mitochondrial phylogeography of rock‐dwelling cichlid fishes reveals evolutionary influence of historical lake level fluctuations of Lake Tanganyika, Africa. Philosophical Transactions of the Royal Society of London. Series B, Biological Sciences, 351, 797–805. https://doi.org/10.1098/rstb.1996.0074 8693021 10.1098/rstb.1996.0074

[ece33860-bib-0053] Wagner, C. E. , & McCune, A. R. (2009). Contrasting patterns of spatial genetic structure in sympatric rock‐dwelling cichlid fishes. Evolution, 63, 1312–1326. https://doi.org/10.1111/j.1558-5646.2009.00612.x 19154384 10.1111/j.1558-5646.2009.00612.x

[ece33860-bib-0054] West, K. , & Michel, E. (2000). The dynamics of endemic diversification: Molecular phylogeny suggests an explosive origin of the thiarid gastropods of Lake Tanganyika. Advances in Ecological Research, 31, 331–354. https://doi.org/10.1016/S0065-2504(00)31018-2

[ece33860-bib-0055] Willing, E.‐M. , Dreyer, C. , & van Oosterhout, C. (2012). Estimates of genetic differentiation measured by F(ST) do not necessarily require large sample sizes when using many SNP markers. PLoS One, 7, e42649 https://doi.org/10.1371/journal.pone.0042649 22905157 10.1371/journal.pone.0042649 PMC3419229

[ece33860-bib-0056] Wilson, A. B. , Glaubrecht, M. , & Meyer, A. (2004). Ancient lakes as evolutionary reservoirs: Evidence from the thalassoid gastropods of Lake Tanganyika. Proceedings Biological Sciences, 271, 529–536. https://doi.org/10.1098/rspb.2003.2624 15129964 10.1098/rspb.2003.2624 PMC1691625

[ece33860-bib-0057] Winkelmann, K. , Rüber, L. , & Genner, M. J. (2017). Lake level fluctuations and divergence of cichlid fish ecomorphs in Lake Tanganyika. Hydrobiologia, 791, 21–34. https://doi.org/10.1007/s10750-016-2839-y

[ece33860-bib-0058] Wright, J. J. , & Bailey, R. M. (2012). Systematic revision of the formerly monotypic genus *Tanganikallabes* (Siluriformes: Clariidae). Zoological Journal of the Linnean Society, 165, 121–142. https://doi.org/10.1111/j.1096-3642.2011.00789.x

[ece33860-bib-0059] Zerbino, D. R. , & Birney, E. (2008). Velvet: Algorithms for de novo short read assembly using de Bruijn graphs. Genome Research, 18, 821–829. https://doi.org/10.1101/gr.074492.107 18349386 10.1101/gr.074492.107 PMC2336801

