# Supplementary material for: Contrasting geographic structure in evolutionarily divergent Lake Tanganyika catfishes
Source: Ecol Evol. 2018 Feb 6;8(5):2688–97. doi: 10.1002/ece3.3860 (PMC5838041; doi:10.1002/ece3.3860)
Supplement: Supplementary file 1 [file ECE3-8-2688-s001.docx]

# Appendix

Table S1 Detailed sampling locations for specimens included in this study

| **Sample No.** | **Species Name** | **Location** | **Latitude** | **Longitude** |
| --- | --- | --- | --- | --- |
| C9 | *Lophiobagrus cyclurus* | Kigoma | -4.880194 | 29.620944 |
| C12 | *Lophiobagrus cyclurus* | Kigoma | -4.880194 | 29.620944 |
| C15 | *Lophiobagrus cyclurus* | Kigoma | -4.886472 | 29.612861 |
| C34 | *Lophiobagrus cyclurus* | Kigoma | -4.880194 | 29.620944 |
| C36 | *Lophiobagrus cyclurus* | Kigoma | -4.880194 | 29.620944 |
| C38 | *Lophiobagrus cyclurus* | Kigoma | -4.880194 | 29.620944 |
| C40 | *Lophiobagrus cyclurus* | Kigoma | -4.880194 | 29.620944 |
| C41 | *Lophiobagrus cyclurus* | Kigoma | -4.880194 | 29.620944 |
| C113 | *Lophiobagrus aquilus* | Sumbu | -8.483306 | 30.467333 |
| C119 | *Lophiobagrus aquilus* | Sumbu | -8.483306 | 30.467333 |
| C134 | *Lophiobagrus cyclurus* | Sumbu | -8.476056 | 30.449444 |
| C141 | *Lophiobagrus cyclurus* | Sumbu | -8.476056 | 30.449444 |
| C161 | *Lophiobagrus cyclurus* | Sumbu | -8.421222 | 30.457778 |
| C166 | *Lophiobagrus cyclurus* | Sumbu | -8.421222 | 30.457778 |
| C171 | *Lophiobagrus cyclurus* | Sumbu | -8.421222 | 30.457778 |
| C172 | *Lophiobagrus cyclurus* | Sumbu | -8.418417 | 30.461417 |
| C174 | *Lophiobagrus cyclurus* | Sumbu | -8.418417 | 30.461417 |
| C175 | *Lophiobagrus cyclurus* | Sumbu | -8.418417 | 30.461417 |
| C227 | *Lophiobagrus cyclurus* | Mpulungu | -8.752972 | 31.084667 |
| C228 | *Lophiobagrus aquilus* | Mpulungu | -8.752972 | 31.084667 |
| C229 | *Lophiobagrus cyclurus* | Mpulungu | -8.752972 | 31.084667 |
| C230 | *Lophiobagrus cyclurus* | Mpulungu | -8.752972 | 31.084667 |
| C236 | *Lophiobagrus aquilus* | Mpulungu | -8.752972 | 31.084667 |
| C237 | *Lophiobagrus cyclurus* | Mpulungu | -8.752972 | 31.084667 |
| C238 | *Lophiobagrus aquilus* | Mpulungu | -8.752972 | 31.084667 |
| C243 | *Lophiobagrus cyclurus* | Mpulungu | -8.753278 | 31.098222 |
| C245 | *Lophiobagrus cyclurus* | Mpulungu | -8.753278 | 31.098222 |
| C246 | *Lophiobagrus cyclurus* | Mpulungu | -8.753278 | 31.098222 |
| C261 | *Lophiobagrus cyclurus* | Mpulungu | -8.753278 | 31.098222 |
| C309 | *Lophiobagrus aquilus* | Mpulungu | -8.797472 | 31.019667 |
| C311 | *Lophiobagrus aquilus* | Mpulungu | -8.797472 | 31.019667 |
| C365 | *Lophiobagrus cyclurus* | Bujumbura Rural | -3.675278 | 29.334167 |
| C373 | *Lophiobagrus cyclurus* | Bujumbura Rural | -3.675278 | 29.334167 |
| C378 | *Lophiobagrus cyclurus* | Bujumbura Rural | -3.675278 | 29.334167 |
| C379 | *Lophiobagrus cyclurus* | Bujumbura Rural | -3.675278 | 29.334167 |
| C383 | *Lophiobagrus cyclurus* | Bujumbura Rural | -3.675278 | 29.334167 |
| C389 | *Lophiobagrus cyclurus* | Bujumbura Rural | -3.675278 | 29.334167 |
| C398 | *Lophiobagrus cyclurus* | Bujumbura Rural | -3.675278 | 29.334167 |
| C406 | *Lophiobagrus cyclurus* | Bujumbura Rural | -3.675278 | 29.334167 |
| S2 | *Synodontis multipunctatus* | Kigoma | -4.886472 | 29.612861 |
| S3 | *Synodontis multipunctatus* | Kigoma | -4.886472 | 29.612861 |
| S28 | *Synodontis multipunctatus* | Kigoma | -4.880194 | 29.620944 |
| S29 | *Synodontis multipunctatus* | Kigoma | -4.880194 | 29.620944 |
| S35 | *Synodontis multipunctatus* | Kigoma | -4.880194 | 29.620944 |
| S38 | *Synodontis multipunctatus* | Kigoma | -4.886472 | 29.612861 |
| S40 | *Synodontis multipunctatus* | Kigoma | -4.886472 | 29.612861 |
| S41 | *Synodontis multipunctatus* | Kigoma | -4.886472 | 29.612861 |
| S160 | *Synodontis multipunctatus* | Mpulungu | -8.752972 | 31.084667 |
| S175 | *Synodontis multipunctatus* | Mpulungu | -8.743667 | 31.059694 |
| S176 | *Synodontis multipunctatus* | Mpulungu | -8.743667 | 31.059694 |
| S180 | *Synodontis multipunctatus* | Mpulungu | -8.743667 | 31.059694 |
| S187 | *Synodontis multipunctatus* | Mpulungu | -8.753278 | 31.098222 |
| S205 | *Synodontis multipunctatus* | Mpulungu | -8.751528 | 31.033222 |
| S221 | *Synodontis multipunctatus* | Mpulungu | -8.751528 | 31.033222 |
| S249 | *Synodontis multipunctatus* | Mpulungu | -8.767139 | 31.099056 |
| S254 | *Synodontis multipunctatus* | Bujumbura Rural | -3.616149 | 29.343914 |
| S257 | *Synodontis multipunctatus* | Bujumbura Rural | -3.616149 | 29.343914 |
| S270 | *Synodontis multipunctatus* | Bujumbura Rural | -3.616149 | 29.343914 |
| S275 | *Synodontis multipunctatus* | Bujumbura Rural | -3.616149 | 29.343914 |
| S282 | *Synodontis multipunctatus* | Bujumbura Rural | -3.616149 | 29.343914 |
| S285 | *Synodontis multipunctatus* | Bujumbura Rural | -3.616149 | 29.343914 |
| S288 | *Synodontis multipunctatus* | Bujumbura Rural | -3.616149 | 29.343914 |
| S290 | *Synodontis multipunctatus* | Bujumbura Rural | -3.616149 | 29.343914 |

# Supplementary Results


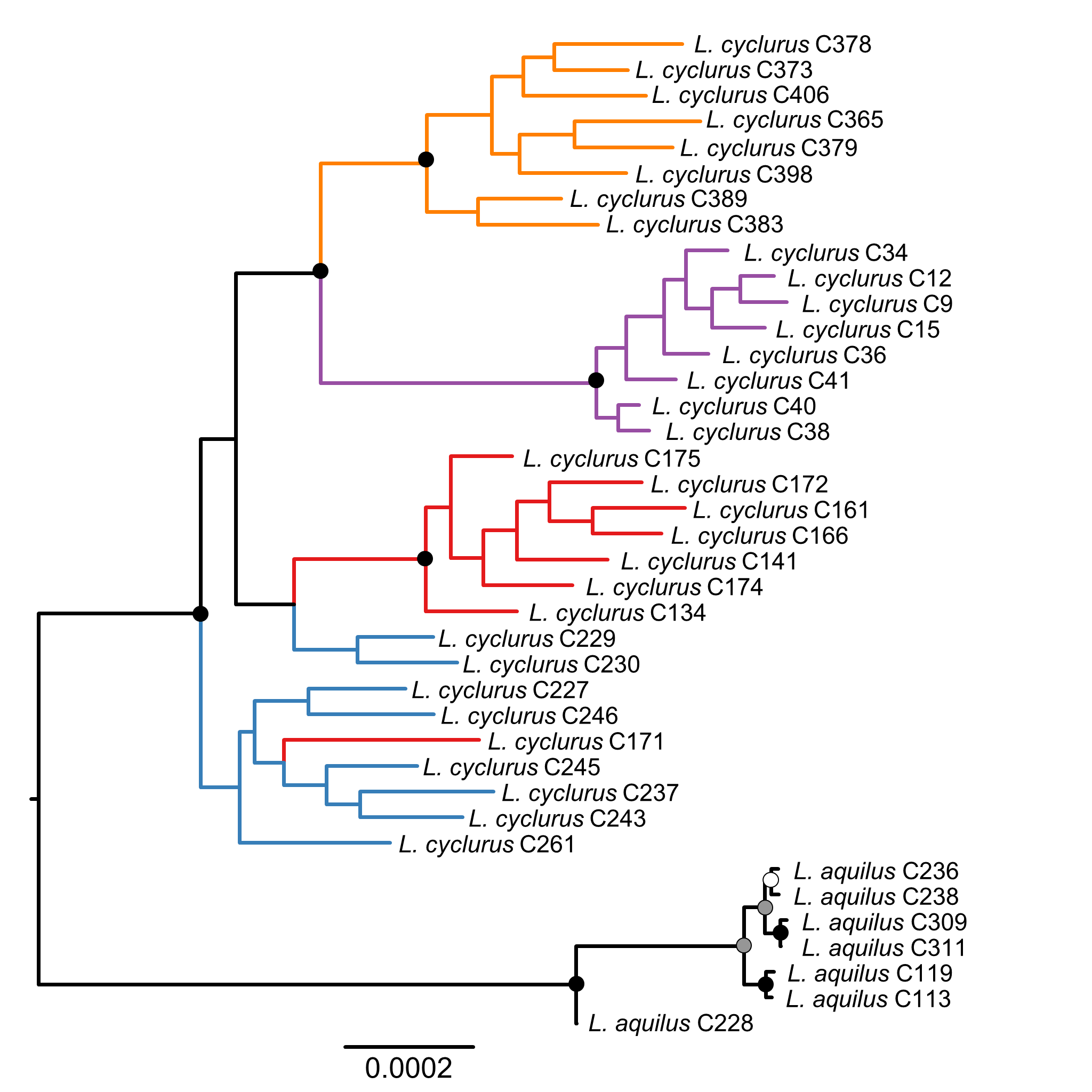


**Figure S1** Maximum-likelihood tree for *Lophiobagrus* with no missing data (bootstrap support: black circles 100%, grey circles >90%, white circles >80%). Colours in the phylogeny depict collection locality, orange- Bujumbura rural, purple- Kigoma, red- Sumbu, blue- Mpulungu.


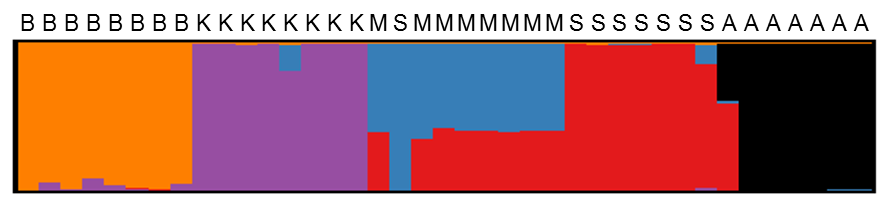


**Figure S2** Structure Plot for *Lophiobagrus* (K=5) with no missing data. Samples are represented in the same order as shown in figure 1 in the main text. The letter above denotes the sampling locality for *Lophiobagrus cyclurus* (B = Bjumbura rural, K = Kigoma, M = Mpulungu, S = Sumbu) with A representing *Lophiobagrus aquilus*.


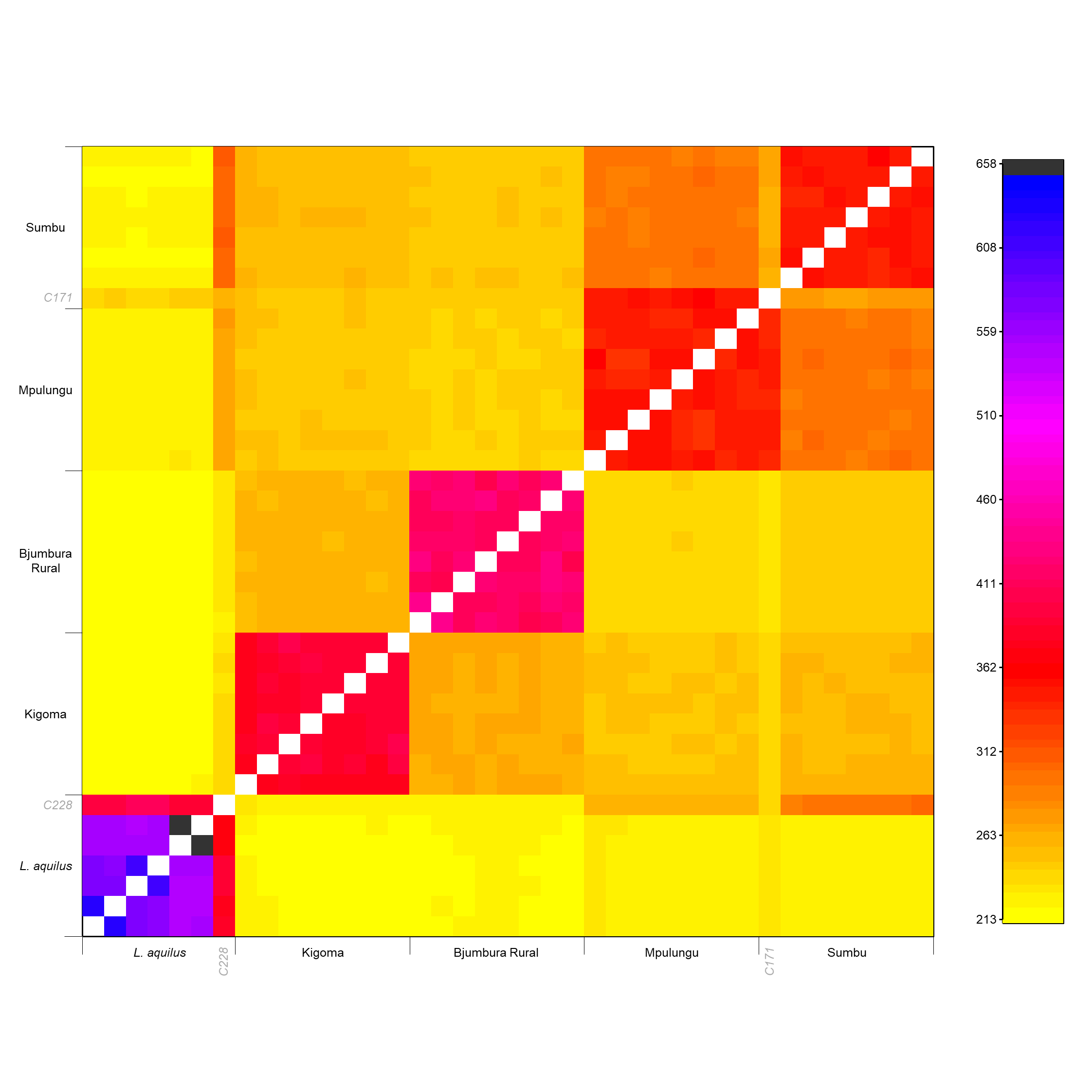


**Figure S3** fineRADstructure Plot for *Lophiobagrus* with no missing data using loci with 1 to 4 SNPs. The admixed *L. aquilus* sample (C228) and C171, which was collected in Sumbu, are annotated.


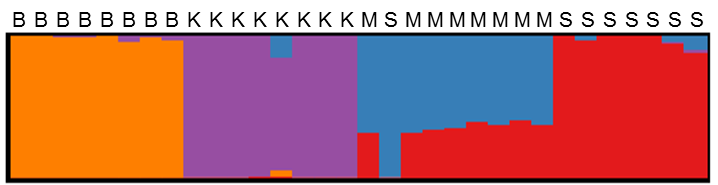


**Figure S4** Structure Plot for *Lophiobagrus cyclurus* only (K=4). Samples are represented in the same order as shown in figure 1 in the main text (with the exception of *L. aquilus* samples). The letter above denotes the sampling locality (B = Bjumbura rural, K = Kigoma, M = Mpulungu, S = Sumbu).


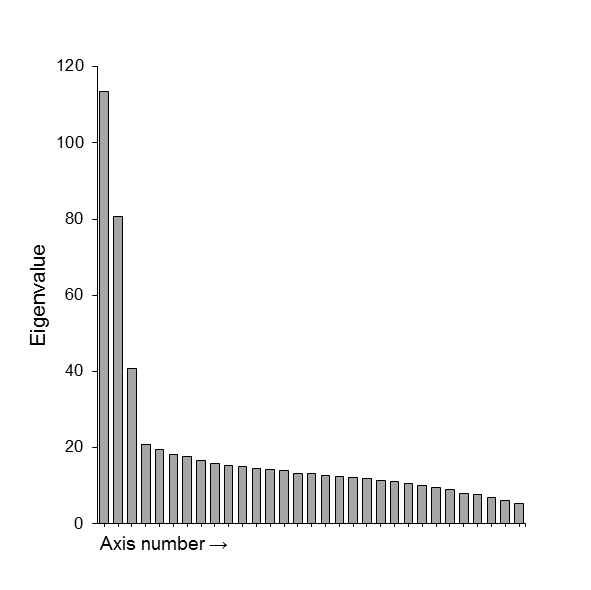

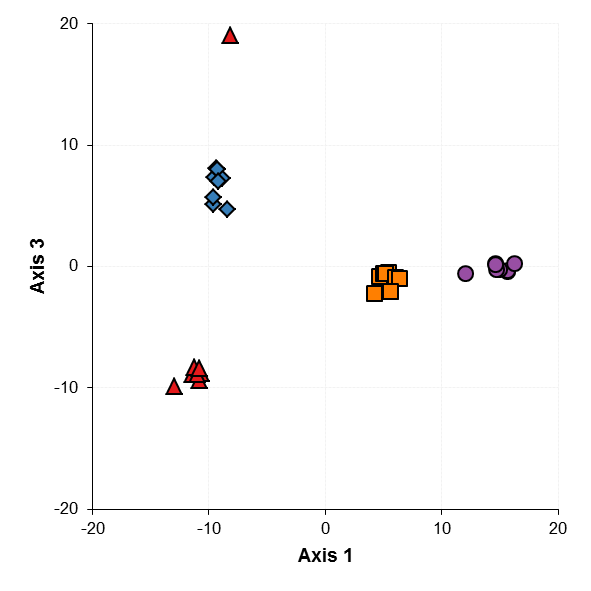

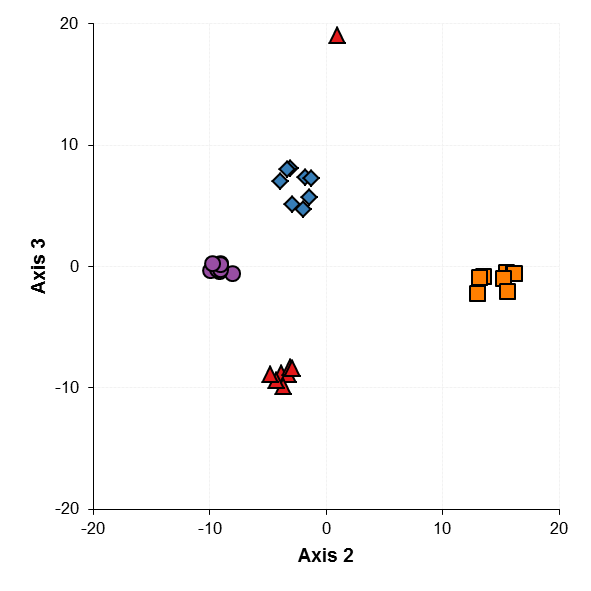

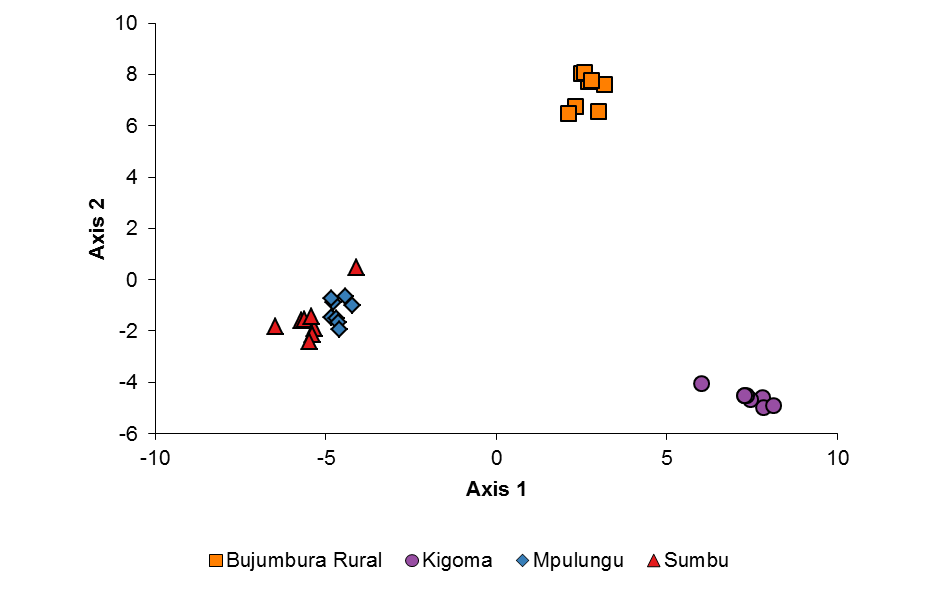


*Lophiobagrus cyclurus*

a)

b)

c)

**Figure S5** Lophiobagrus cyclurus a) Scree plot showing eigenvalues for all PCA axes, b) PCA plot for Axis 1 vs Axis 3, c) PCA plot for Axis 2 vs Axis 3

Table S2 Nominal D Statistics for *L. cyclurus* (without sample C228) with mean (and standard deviation) of nominal D value from 1,000 random subsamples of the dataset at each percentage coverage. The taxa in bold show evidence of admixture based on the D-statistic values.

| Tree topology | Overall  D statistic | D statistic ± standard deviation at subsampling level | | | | |
| --- | --- | --- | --- | --- | --- | --- |
|  |  | 99% | 95% | 90% | 80% | 70% |
| ┌── Burundi  ┌─│  ┌─│ └── **Kigoma**  │ └──── **Mpulungu**  └────── *L. aquilus* | 0.0673 | 0.0673 (±2.1 × 10⁻⁵) | 0.0673 (±5.1 × 10⁻⁵) | 0.0672 (±9.1 × 10⁻⁵) | 0.0671 (±1.2 × 10⁻⁴) | 0.0672 (±1.1 × 10⁻⁴) |
| ┌── Burundi  ┌─│  ┌─│ └── **Kigoma**  │ └──── **Sumbu**  └────── *L. aquilus* | 0.0736 | 0.0736 (±1.7 × 10⁻⁵) | 0.0735 (±3.2 × 10⁻⁵) | 0.0735 (±5.4 × 10⁻⁵) | 0.0737 (±1.5 × 10⁻⁴) | 0.0734 (±1.7 × 10⁻⁴) |
| ┌── Mpulungu  ┌─│  ┌─│ └── **Sumbu**  │ └──── **Burundi**  └────── *L. aquilus* | 0.0556 | 0.0557 (±1.7 × 10⁻⁵) | 0.0557 (±3.2 × 10⁻⁵) | 0.0556 (±6.6 × 10⁻⁵) | 0.0556 (±5.6 × 10⁻⁵) | 0.0556 (±1.1 × 10⁻⁴) |
| ┌── Mpulungu  ┌─│  ┌─│ └── **Sumbu**  │ └──── **Kigoma**  └────── *L. aquilus* | 0.0628 | 0.0628 (±1.8 × 10⁻⁵) | 0.0628 (±4.7 × 10⁻⁵) | 0.0628 (±4.7 × 10⁻⁵) | 0.0628 (±1.2 × 10⁻⁴) | 0.0628 (±1.2 × 10⁻⁴) |


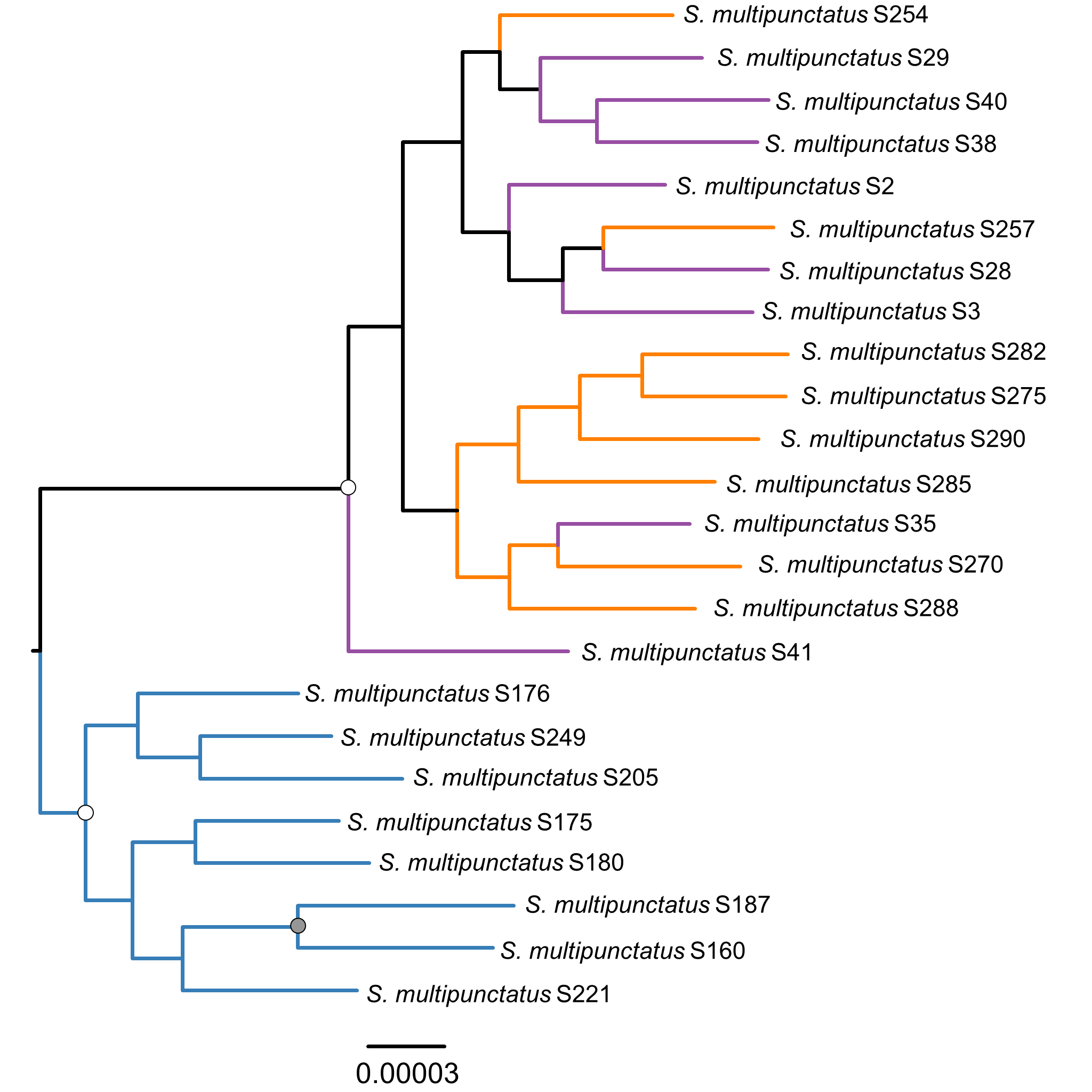


**Figure S6** Maximum-likelihood tree for *Synodontis multipunctatus* with no missing data (bootstrap support: black circles 100%, grey circles >90%, white circles >80%). Colours in the phylogeny depict collection locality, orange- Bujumbura rural, purple- Kigoma, red- Sumbu, blue- Mpulungu.


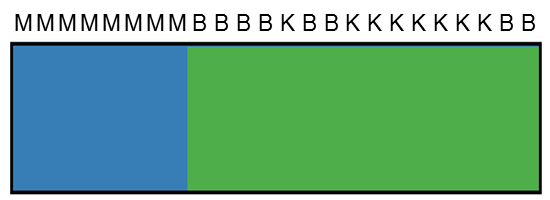


**Figure S7** Structure Plot for *Synodontis multipunctatus* (K=2) with no missing data. Samples are represented in the same order as shown in figure 1 in the main text. The letter above denotes the sampling locality (B = Bjumbura rural, K = Kigoma, M = Mpulungu).


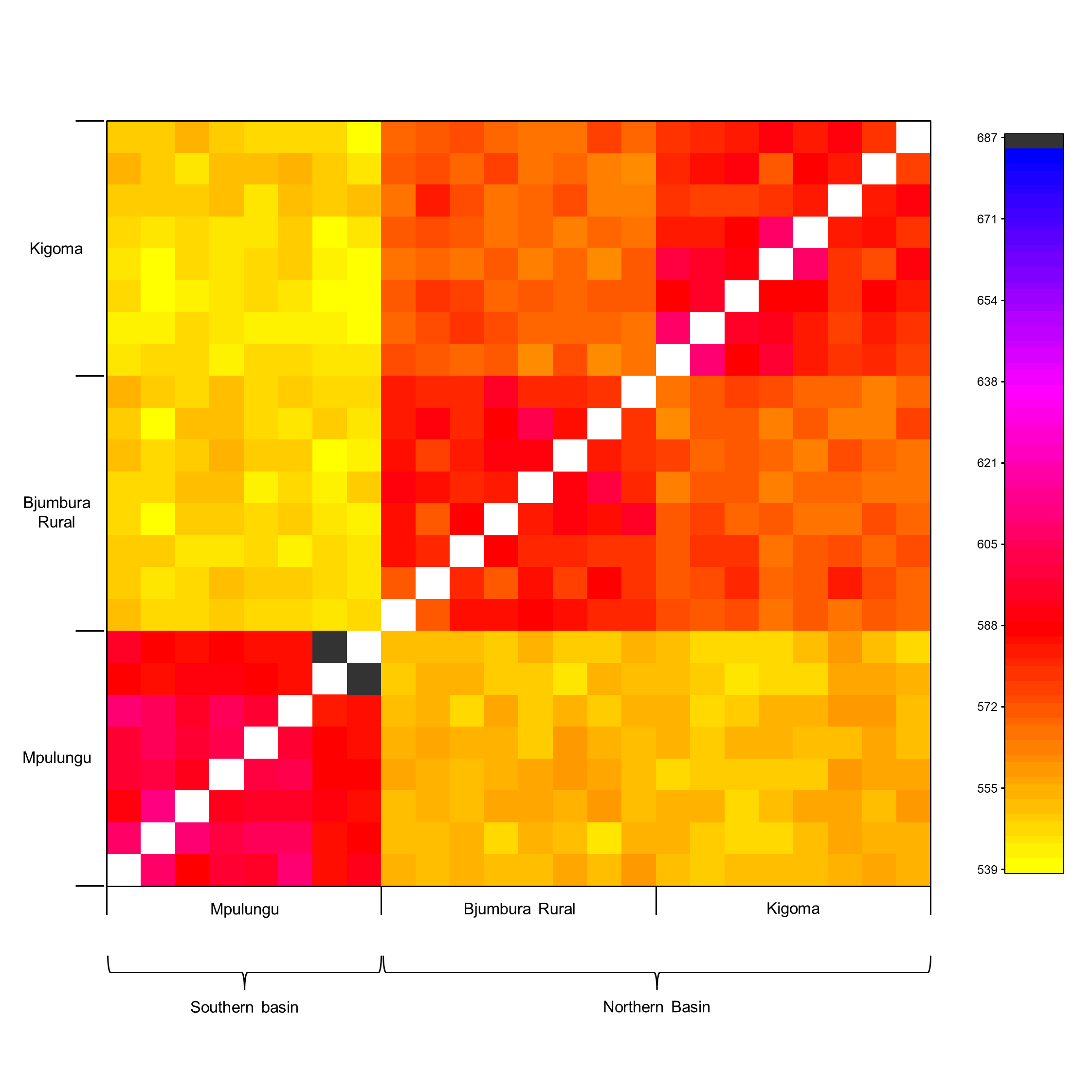


**Figure S8** fineRADstructure Plot for *Synodontis multipunctatus* with no missing data using loci with 1 to 4 SNPs.

*Synodontis multipunctatus*

**Figure S9** Scree plot showing eigenvalues for all PCA axes for *Synodontis multipunctatus*


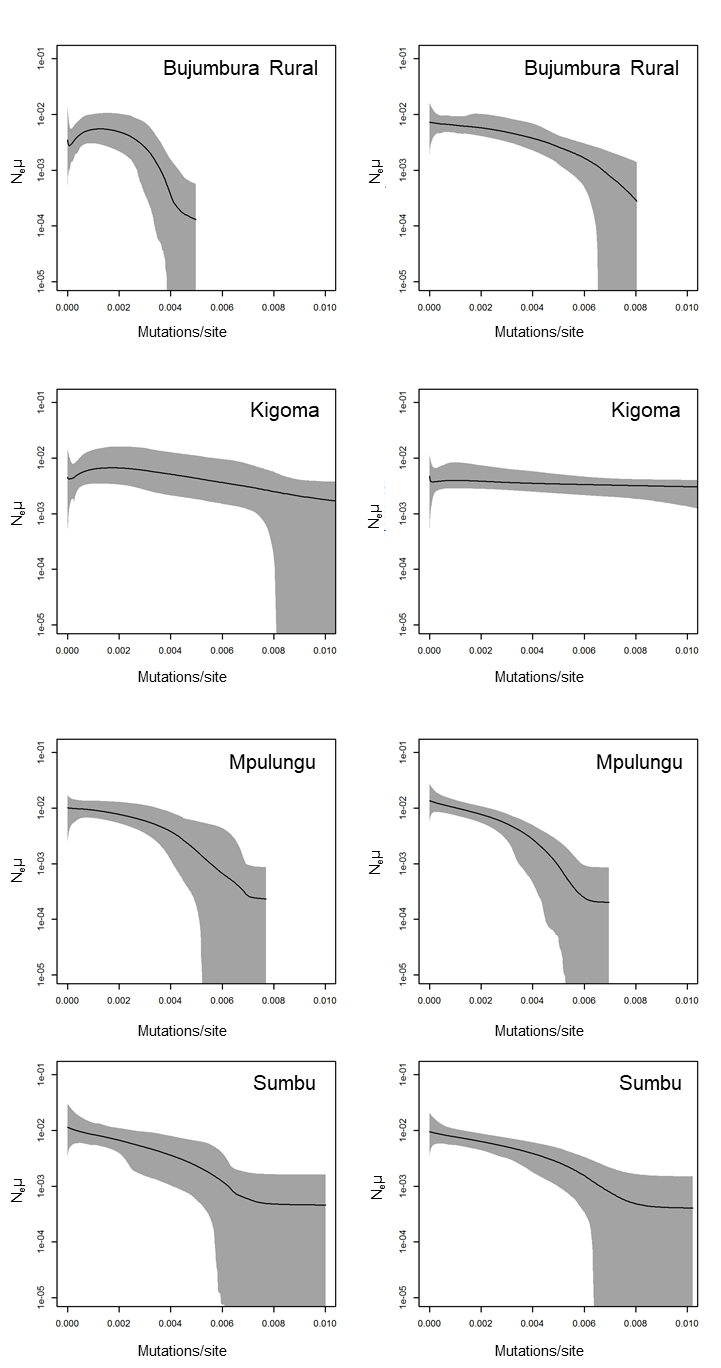


**Figure S10** Bayesian skyline plots for the remaining subsamples of *Lophiobagrus cyclurus*


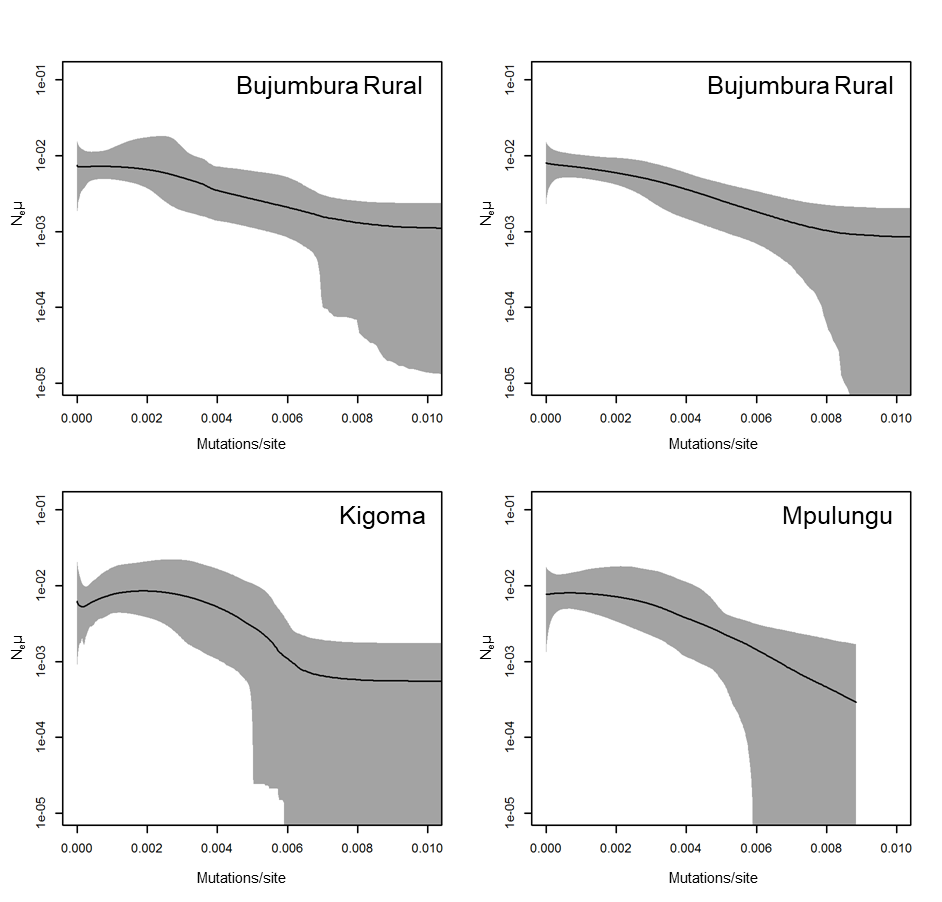


**Figure S11** Bayesian skyline plots for the remaining subsamples of *Synodontis multipunctatus*
